# Statistical Properties of Correlated Semiclassical Bands in Tight-Binding Small-World Networks

**DOI:** 10.3390/e27040420

**Published:** 2025-04-12

**Authors:** Natalya Almazova, Giorgos P. Tsironis, Efthimios Kaxiras

**Affiliations:** 1 Institute of Theoretical and Computational Physics, Department of Physics, University of Crete, 71003 Heraklion, Greece; gts@physics.uoc.gr; 2 John A. Paulson School of Engineering and Applied Sciences, Harvard University, Cambridge, MA 02138, USA; kaxiras@g.harvard.edu; 3 Department of Physics, Harvard University, Cambridge, MA 02138, USA

**Keywords:** discrete nonlinear Schrödinger equation, small-world networks, long-range interaction, stationary states, density of states

## Abstract

Linear tight-binding models with long-range interactions and small-world geometry have a broad energy spectrum in the nearest neighbor coupling limit, while the spectrum becomes narrow in the fully connected limit due to the emergence of flat bands. A transition to a Wigner-like density of states appears at a low fraction of long-range bonds. Adding nonlinearity to the model introduces correlations among the stationary states, while multiple new states are generated as a result of the nonlinearity. In this work, we study the effect of band correlations on the local density of states for small-world networks as a function of the number of long-range bonds. We find that close to the nearest neighbor limit, the onset of correlations shifts the nonlinear density of states towards the band edge of the spectrum. Close to the opposite limit of the fully connected model, the band collapses in the band center, accompanied by a large increase in the new states induced by the nonlinearity. While in both limits the effect of correlations is to flatten the band, close to the mean field fully connected limit, the states are correlated and generally have distinct localized features. These effects may have implications for the dynamics of electrons in two-dimensional moiré structures and the onset of superconductivity in these systems.

## 1. Introduction

The interplay of disorder with quantum particle correlations plays an important role in the physics of systems with strong interactions and has significant ramifications for the properties of novel materials. Disorder typically induces localization, which may be enhanced through particle interactions. On the other hand, the very presence of disorder-induced localized states that are close in energy may lead to new behavior in the transport properties. A case in point is the behavior of twisted bilayer graphene (tBLG), where the twist-induced macroscopic-scale features, the moiré pattern, dramatically affect the electronic conduction properties, leading to superconductivity [1,2]. The transition to a superconducting state appears to be related to flat bands in the spectrum in tBLG, the presence of which was predicted by theory [3] long before the experimental observation of correlated behavior. The physics of twisted moiré materials turned out to be very rich and interesting, as it involves the interplay between macroscopic features and strong electron interactions; insights into the origin of this behavior from different perspectives are thus very useful.

The small-world tight-binding model with cubic nonlinearity, as in the discrete nonlinear Schrödinger system [4] (SW-DNLS), offers a simple framework for studying the interplay between long-range randomness with particle correlations. In this model, a set of long-range bonds are randomly added to the existing nearest neighbor interactions, representing the effective randomness, while the particle correlations are induced by localized nonlinearity. In recent work [5], we focused on the tight-binding small-world model in the linear regime and found that the electron dynamics have two distinct limits: the first limit is close to the nearest neighbor regime, while the second limit is close to the fully connected system. While the Density Of States (DOS) retains the typical square-root singular shape in the former limit, it reduces to a quasi-flat band in the latter limit. The spectral shape changes drastically, with only a few percent of additional long-range bonds, and quickly assumes a Wigner-like semicircular shape.

The complex spectral characteristics for the simple small-world linear model suggest that inclusion of electronic correlations may have interesting ramifications for these dynamics, with possible connections to the behavior of moiré materials. The correlations can be included through the DNLS equation, with the nonlinear term arising from effective interactions that represent eliminated degrees of freedom, which leads to electron correlations. The SW-DNLS model thus contains all three ingredients; namely, disorder, long-range (LR) interactions, and nonlinearity-induced correlations. We note that its linear limit is well understood both analytically and numerically. Consequently, it is useful to begin analysis from the linear regime, add nonlinearity to the problem, and investigate its role in the spectrum and dynamics.

The structure of this paper is as follows: In Section 2, we introduce the mathematical model and discuss the various parameters and the resulting physics; in Section 3 we present an extended set of numerical results for the nonlinear DOS distribution for various parameter values; finally, in Section 4, we present the conclusions of our numerical studies. While the basic work presented here is numerical, it is based on earlier analytical as well as numerical studies that focused on smaller DNLS units that may be studied analytically, as well as extended tight-binding configurations in one dimension.

## 2. The Nonlinear Small-World Network Model

**Figure 1:**
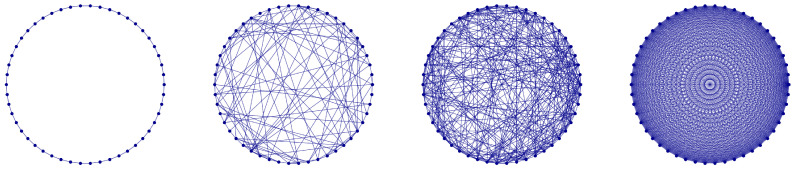
Schematic representation of the one-dimensional small-world system with periodic boundary conditions: the number of neighbors ranges from the nearest neighbor (NN) only (left image), to few or many random connections (two middle images), to the fully-connected, mean field (MF) limit system (right image).

We introduce an SW-DNLS system as an extension of the linear small-world system that was studied earlier [5,6]. We consider a periodic lattice with *n* sites that has nearest neighbor coupling *V*, as well as a number of random long-range bonds with coupling strength *W*, as shown schematically in Figure 1. In this lattice, we also add local on-site nonlinear terms that induce spatial interaction in the electronic probability distribution. This model, in a similar form, has been used to find biologically relevant local modes induced by nonlinearity in proteins [7]. Although the model is quite general, we will focus on specific aspects that may be of possible significance to twisted layered materials. Specifically, we endeavor to find how the correlations induced by the local nonlinearity affect the stationary spectrum of the system.

### 2.1. The Dynamical Equation

The DNLS equation is used to describe the behavior of general systems in condensed matter, chemistry, and optical systems that exhibit self-trapping phenomena [8,9,10,11,12]. While the equation is dynamical, it has a form that leads to a direct evaluation of a special set of solutions that are stationary [4,13]. These solutions, although not the most general dynamical solutions of the equation, are particularly useful, since they correspond to the eigensolutions of the corresponding linear problem. In addition to the general DNLS model, the stationary states of the DNLS equation with cubic-quintic nonlinearity were analyzed in [14], while they were also used to model the behavior of a group of interconnected nonlinear anharmonic oscillators and to explore nonlinear localization effects [15]. For simplicity, we assume all system sites have the same energy, which is set to zero. The SW-DNLS equation has, in addition to the standard nearest neighbor bonds with strength *V*, additional long-range bonds with strength *W*; we express this as follows:

(1) $$\begin{array}{cc} i\frac{\partial \psi_{j}}{\partial t} & =V\left(\psi_{j+1}+\psi_{j-1}\right)+W\sum_{k=2}^{(n-1)/2}g_{j,j+k}\left(\psi_{j+k}+\psi_{j-k}\right)-\epsilon {\left|\psi_{j}\right|}^{2}\psi_{j} \end{array}$$

with $\psi_{j}\equiv \psi_{j}\left(t\right)$ being the probability amplitude used to find a particle at site *j*. It is useful to designate as $\psi =col(\psi_{1},\psi_{2},\ldots ,\psi_{n})$ the vector of complex-valued modes in the site representation and note that $|\psi_{j}{|}^{2}$ is the probability of detecting a particle in the location j=1,2,…,n at time *t*. We denote the nearest neighbor matrix element overlap, or nearest neighbor bond strength by *V*. The long-range interaction part of the model is included through the second term on the right-hand side of Equation (1); this term is proportional to the parameter *W* that controls the strength of the long-range bonds. The long-range bonds are assumed to be proportional to the same constant *W*, while the presence or absence of a long-range bond is determined by the function $g_{j,j+k}$, which is taken to be 1 if there is a long-range bond between sites *j* and j+k, or 0 if there is no such connection. For simplicity of notation, we introduce γ=W/V, the parameter that controls the relative strength of the long-range bonds to that of nearest neighbor couplings. We also use *V* to rescale the time *t* (t→Vt) and the nonlinear parameter ϵ by introducing χ=ϵ/V. In the rescaled variables, the SW-DNLS reads

(2) $$\begin{array}{cc} i\frac{\partial \psi_{j}}{\partial t} & =\left(\psi_{j+1}+\psi_{j-1}\right)+\gamma \sum_{k=2}^{(n-1)/2}g_{j,j+k}\left(\psi_{j+k}+\psi_{j-k}\right)-\chi {\left|\psi_{j}\right|}^{2}\psi_{j} \end{array}$$

The energy or frequency scale is then measured in units of *V*. Equation (2) can be expressed in a matrix form, combining short-range and long-range interactions into a single component [4]:

(3) $$\begin{array}{c} i\frac{\partial \psi }{\partial t}=M\psi -{\chi D(|\psi |}^{2})\psi \end{array}$$

where ${D(|\psi |}^{2})=diag(|\psi_{1}{|}^{2},|\psi_{2}{|}^{2},|\psi_{3}{|}^{2},...,|\psi_{n}{|}^{2})$ is a n×n diagonal matrix representing the nonlinear term, which includes anharmonicity. $M=[m_{ij}]$ is a n×n linear coupling matrix between the sites. *M* should be real and symmetric. There are two conserved quantities in the DNLS, Equation (3), viz. the energy H and the particle number N, which are given by the expressions

(4) $$\begin{array}{c} H=-\frac{1}{2}\chi \sum_{i=1}^{n}|\psi_{i}{|}^{4}-\sum_{i,j}m_{ij}\psi_{i}^{*}\psi_{j}\;,\;N=\sum_{i=1}^{n}{\left|\psi_{i}\right|}^{2}\;. \end{array}$$

These quantities are also conserved in the SW-DNLS equation and, as a result, we can use the approach of Eilbeck et al. [4] to analyze the properties of this more generalized model. Regarding physics, we note that the long-range DNLS model on which we focus may give significant clues about the role long-range interactions play in quantum systems, while, at the same time, incorporating the effects of disorder. The latter, in the present approach, is introduced through the small-world nature of the bonds. It is worth commenting that in moiré materials, the twist angle process introduces longer-range interactions in the lattice planes; this fact may be in part responsible for the onset of flat bands. Studying, then, the role of nonlinearity in a long-range tight-binding setting helps understand the induced correlations in the moiré states.

### 2.2. Stationary Solutions

A general time-dependent solution of the SW-DNLS equation is not available, except for some special cases [12,16]. Stationary solutions are more readily accessible, representing in some sense an extension of the linear eigenstates in the presence of nonlinearity. A stationary analysis gives insight into the multiplicity of modes present in the complex correlated network. In order to obtain the stationary solutions of the DNLS model, we use the ansatz [8]

(5) $$\begin{array}{c} \psi (t)=A\cdot \exp(i\omega t), \end{array}$$

where *A* is a real and time-independent vector and ω is the circular frequency of the corresponding mode. Substituting the expression of Equation (5) into the DNLS Equation (3) yields a nonlinear algebraic eigenvalue problem:

(6) $$\begin{array}{c} -\omega A+\chi D(|A{|}^{2})A+MA=0, \end{array}$$

with the sum of probabilities being equal to one, viz. $\sum_{j}{\left|A_{j}\right|}^{2}=1$. The Equation (6) presents a form of a non-linear eigenvalue-type problem that results in a SW-DNLS special solution characterized by frequency ω and amplitude *A*. To determine each solution, we use a constant system size *n* and a given nonlinearity parameter χ. The symmetry of *M* ensures that all solutions are real.

When the nonlinearity parameter is absent, i.e., for χ=0, the Equation (6) reduces to the linear eigenvalue problem MA=ωA; the frequencies ω are now true eigenenergies of the system and can be determined through the standard determinant equation det|MA−ωA|=0 [5]. When the nonlinearity is “turned on”, these linear eigenstates become stationary states of the nonlinear system that initially continue, i.e., or small nonlinearity, analytically from the χ=0 values. For a certain initial range of the nonlinearity parameter, the system is quasi-linear and the frequencies of the modes simply change in value. However, for larger values of χ, we observe abrupt changes in the states that are accompanied by the onset of new states; this feature has been observed in both the stationary problem [4] and in the dynamics [17]. In the present extension of the analysis in the SW-DNLS model, we use a numerical approach in order to evaluate the frequency ω and the corresponding amplitudes of the states. Specifically, we employ standard continuation techniques that have been successfully used in similar problems [13,18,19,20,21,22].

### 2.3. Small-World Network Geometry

The concept and geometry of Small-World Networks (SWN) [23] has been applied to the dynamics of the DNLS model [6,24]. SWN’s are seen to be ubiquitous in real-world systems, ranging from social networks [23,25], to biological neural networks [26], transportation networks [27,28,29], network neuroscience [30], as well as machine learning [31,32,33]. For our study, we form a SWN as follows: We start from a one-dimensional nearest neighbor lattice of *n* sites that we shape into the form of a ring; i.e., we assume periodic boundary conditions. Subsequently, we introduce randomly long-range bonds joining distant non-nearest neighbor sites. We take the bond strength of the nearest neighbors to be 1, while for the long-range bonds, it takes a value γ. The SWN thus has a variable number of additional long-range bonds; this number is used as an additional parameter in the study.

After the geometry is set, we numerically solve Equation (6) and determine the frequencies and spatial distribution of the corresponding states. Since the model is random, we repeat the procedure for several random bond distributions and obtain statistics of the frequencies of the DNLS equation on the SWN. This analysis provides useful nonlinear spectral features that ultimately give clues about the dependence of dynamics on nonlinearity and random long-range bonds. The SWN geometry has two limits: the Nearest Neighbor (NN) limit, and the Fully Connected or Mean Field (MF) limit, where every site in the system is connected to every other site. All intermediate cases involve a given number of long-range random connections between sites in the system, as illustrated in Figure 1.

The matrix elements of *M*, which are denoted as $m_{i,j}$, take the value 1 for the nearest neighbor interaction, while the long-range connections have strength γ when present or zero if no long-range bond is present. In the NN limit, the coupling tight-binding matrix is expressed as follows:

(7) $$\begin{array}{c} M_{NN}=\left[\begin{array}{cccccc} 0 & 1 & 0 & \cdots & 0 & 1\\ 1 & 0 & 1 & \cdots & 0 & 0\\ 0 & 1 & 0 & \cdots & 0 & 0\\ \vdots & \vdots & \vdots & \ddots & \vdots & \vdots \\ 0 & 0 & 0 & \cdots & 0 & 1\\ 1 & 0 & 0 & \cdots & 1 & 0 \end{array}\right], \end{array}$$

with the two values on the anti-diagonal imposing the periodic boundary conditions. In the MF limit, on the other hand, for γ=1, the coupling matrix takes the following simple form:

(8) $$\begin{array}{c} M_{MF}=\left[\begin{array}{cccccc} 0 & 1 & 1 & \cdots & 1 & 1\\ 1 & 0 & 1 & \cdots & 1 & 1\\ 1 & 1 & 0 & \cdots & 1 & 1\\ \vdots & \vdots & \vdots & \ddots & \vdots & \vdots \\ 1 & 1 & 1 & \cdots & 0 & 1\\ 1 & 1 & 1 & \cdots & 1 & 0 \end{array}\right]. \end{array}$$

We note that, in this extreme limit, all sites couple to every other site with the same strength, leading to a highly degenerate system [5].

In order to investigate all intermediate SWN configurations, we introduce the parameter *B* that measures the number of long-range couplings between the system sites [5]. For a one-dimensional lattice with *n* sites in a ring configuration, we have *n* number of NN bonds. On the other hand, in the MF limit, we have the largest number of long-range bonds equal to

(9) $$\begin{array}{c} B_{\max}=\frac{n(n-1)}{2}-n. \end{array}$$

Thus, while in the NN limit the number of long-range random connections is B=0, in the opposite MF limit, this is equal to $B=B_{\max}$. It is useful to introduce $B_{\mathrm{per}}=\left(B/B_{\max}\right)\times 100$; this quantity describes the number of long-range connections in the lattice as a percentage of the number of long-range connections.

## 3. Nonlinear Small-World Network Spectra

We conducted an extensive numerical study in order to investigate the nonlinear spectral behavior of the SW-DNLS model. We wished to understand the stationary behavior of the system as a function of the nonlinearity parameter χ, while, at the same time, the random geometry of the system varied. The additional parameter of the strength of the long-range bonds γ was also varied.

### 3.1. Frequency Dependence on the Nonlinearity Parameter χ

**Figure 2:**
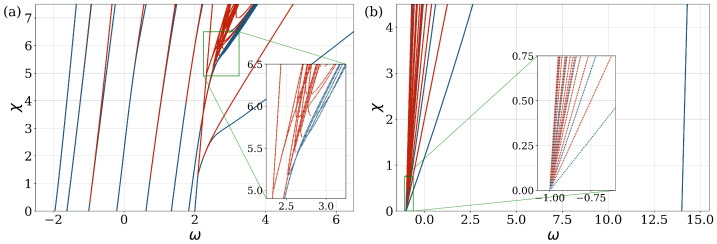
Frequency ω (abscissa) as a function of the nonlinear parameter χ for the limiting cases of the small-world network geometry: (**a**) $B_{\mathrm{per}}=0\%$ (Nearest Neighbor limit), and (**b**) $B_{\mathrm{per}}=100\%$ (fully connected Mean Field limit). The number of sites n=15. The color blue (red) denotes stable (unstable) states.

The first aim was to determine the behavior of the two extreme cases of the SWN geometry, NN and MF (fully connected), represented by $B_{\mathrm{per}}=0\%$ and $B_{\mathrm{per}}=100\%$, respectively, by varying the nonlinearity parameter of the system. The spectrum of the frequencies of the stationary states, as well as the corresponding stationary states of Equation (6), were computed numerically as a function of the nonlinear parameter χ using a continuation method [13,21]. For χ=0 (linear case), the solutions could be determined analytically. The eigenvalues and eigenstates of the linear case were used as initial values, in order to find the next frequencies and stationary states with a slightly increased χ parameter. This iterative process was continued until the desired χ value was reached. Figure 2 illustrates the evolution of the system in χ-space as a function of the nonlinear parameters for the two limiting cases of the system with n=15 sites. The numerical calculation details are provided in Appendix A.

The analysis revealed the presence of points where new solution branches emerged or existing branches split. These bifurcations are characteristic signatures of nonlinear systems and signify transitions between qualitatively distinct stationary states. The system generated new states as the nonlinearity increased, even with a relatively small number of additional long-range connections. In the NN limit, the departure from the linear eigenstates due to nonlinearity showed new states as the value of nonlinearity increased [4]. Variations in system parameters, such as nonlinearity strength, coupling constant, or percentage of the LR connections, can alter the stability of the solution branches. These parameter changes may induce stationary state changes, leading to the emergence, disappearance, or exchange of stability between different solution branches. In Figure 2, we show both stable (blue) and unstable (red) states. When the system size is an even number $n=n_{2k}$, ω is distributed symmetrically between −2 and 2, and each of the frequencies is degenerate for the case with zero nonlinearity. However, if $n=n_{2k+1}$, the symmetric distribution breaks, but the degeneracy stays in place, the states n/2−1 are degenerate and one nondegenerate. The degeneracy appears because of the translational symmetry of the matrix *M* Equation (7) [34]. By calculating a frequency diagram, we found that the nonlinearity affected the degenerate stationary states, leading to new branches of solutions. These cause an asymmetric shift of the frequency, eliminating degeneracy [35]. In the context of a small nonlinearity, the system manifests two degenerate stable states. However, as the nonlinearity increases, these degenerate states undergo a split, resulting in two distinct solutions. One branch remains stable, while the other becomes unstable; thus the stability of branches changes.

In the MF limit, for χ=0, the spectrum of the matrix *M* Equation (8) consists of n−1 degenerate eigenstates with an eigenvalue of ω=−1, and a single eigenvalue with a value of ω=n−1, the Peron–Frobenius state [6]. The stationary state spectrum becomes more dispersed as χ increases. Small nonlinearity removes degeneracy and all branches spread around a certain region. When all states are connected (MF limit), the impact of the nonlinear parameter is averaged over the system [16]. More branches become unstable as the nonlinearity increases, but some of them maintain stability at a large nonlinearity. The branch at ω=n−1 stays stable for the entire nonlinearity regime.

### 3.2. Random Connections with Equal Strength Bonds

We first consider the case where all bonds, NN as well as long-range, have the same strength. Although this is a rather extreme limit, it nevertheless eliminates one parameter from the problem, viz. γ, and this makes the understanding of the mutual effects of nonlinearity with SWN-order more transparent. We thus analyzed the spectral representation of a system consisting of n=100 sites, with a fixed nearest neighbor interaction strength, as well as γ=1.0. To obtain solutions for the stationary states, we numerically computed the stationary states of Equation (6) for a given nonlinear parameter, incorporating random long-range (LR) couplings that formed the coupling matrix *M*. Two cases were selected for illustration, $B_{\mathrm{per}}=0.06\%$ (close to the NN limit) $B_{\mathrm{per}}=97.0\%$ (close to the MF limit).

**Figure 3:**
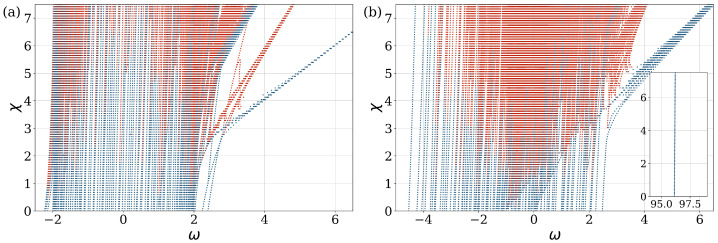
Mode frequency ω as a function of nonlinear parameters χ for cases of the small-world network geometry, for different numbers of LR bonds: (**a**) $B_{\mathrm{per}}=0.06\%$, and (**b**) $B_{\mathrm{per}}=97.0\%$. The inset in (**b**) shows the position of the isolated state, as in the corresponding linear problem [5]. The number of sites is n=100. The color blue (red) is used to denote stable (unstable) states.

In Figure 3, we present the frequencies of the various nonlinear modes as a function of nonlinearity χ, while, at the same time, portraying their stability. In Figure 3a, we show the behavior of the system for $B_{\mathrm{per}}=0.06\%$. All the frequencies are distributed in a range of [−2,2]. Frequency degeneracy is lifted for the stable and unstable solutions, induced by the strong influence of nonlinearity in the breaking of the translational symmetry of the system. As the nonlinearity increases, new unstable modes emerge. The number of new solutions increases at the bond edges, indicating that stationary states with short wavelengths are dominant. The SWN topology modifies the frequency distribution. For $B_{\mathrm{per}}=97.01\%$, the system displays a notable increase in the number of new frequencies at its center, as seen in Figure 3b The stationary states shown in the figure are distributed in the range between −4.5 and 2.5. This range is non-symmetric due to the relatively small size of the system. As the system size increases to sizes larger than approximately 1000 sites, the frequency range becomes symmetric, as in the linear case. Indeed, the density of states (DOS) in [5] for the larger system size, e.g., 5000 sites in the linear, is seen to be a symmetric distribution with zero mean value. Small, finite systems have a certain sensitivity to the frequency distribution of stationary states and lead to asymmetric distributions. Within the limit of an infinite system, this distribution becomes symmetric. We also note that more unstable branches are located in the central part of the frequency diagram. The presence of nonlinear terms in the system’s governing equations introduces complex interactions between variables. In contrast to the linear case, these interactions permit the existence of multiple solutions or behaviors for a given set of parameters. As the values of the parameters χ, $B_{\mathrm{per}}$ and γ change, they may reach a critical threshold, resulting in a fundamental alteration of the system’s equilibrium structure or stability properties.

**Figure 4:**
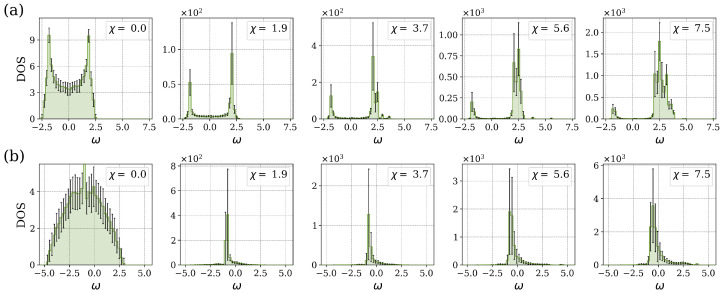
The DOS as a function of frequency ω for different values of the nonlinear parameter χ and the percentage of LR connections: (**a**) $B_{\mathrm{per}}=0.06\%$ and (**b**) $B_{\mathrm{per}}=97.01\%$. In both cases, the LR interaction strength is γ=1.0. The error bars represent the standard deviation.

In order to provide a more comprehensive representation of the system’s spectral properties for varying percentages of LR bond interactions, we computed the DOS as a function of the frequency of stationary states. We averaged the DOS over 50 randomly generated instances. Figure 4 illustrates the density of states for various values of the nonlinear parameter χ. The system with n=100 sites has a maximum of $B_{max}=4850$ LR connections. The presence of random long-range connections significantly influences the spectral characteristics of the system. Specifically, there is a transition from a square-root singularity, which is typical of NN coupling, to a semicircular shape characteristic of the MF limit. An analogous behavior was also observed in the corresponding linear model [5].

The transition from the square-root singularity to a semicircular distribution of the spectrum occurs at a relatively low percentage of additional LR connections, approximately $B_{\mathrm{per}}^{c}=3\%$ of the maximum number of LF bonds for the n=100 system. When the percentage of additional LR bonds is below the critical value $B_{\mathrm{per}}^{c}$, the spectrum exhibits two local maxima located near ±2. For the system with a low percentage of additional LR bonds ($B_{\mathrm{per}}=0.06\%$), the distribution of stationary states exhibits a shift to the right bond edge of the spectrum. The nonlinear component of the system leads to the appearance of split solutions, which result in an asymmetric distribution of the density of states. Frequencies greater than 7.5 are not shown in Figure 4a. The standard deviation of the DOS distribution is shown by error bars. The increased standard deviation at higher levels of nonlinearity indicates that random long-range connections impact the system in different ways, while maintaining the underlying structural characteristics. Near the MF limit, Figure 4b ($B_{\mathrm{per}}=97.01\%$), the frequency spectrum ω indicates a concentration of stationary states in the center of the distribution around zero in the range [−4.5,2.5], and the DOS has a semicircular shape for the system with n=100 sites. Furthermore, some frequencies remain close to the value n−1.

**Figure 5:**
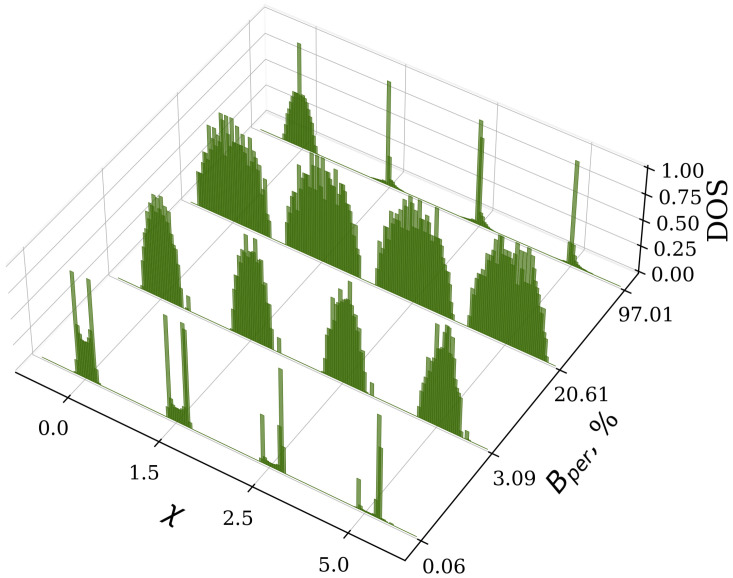
The distribution of DOS as a function of the nonlinear parameter χ and percentage of LR connections $B_{\mathrm{per}}$, for γ=1.0. Each DOS has been normalized to a value of 1 for comparison.

To show the transformation of the distribution of the DOS as a function of the nonlinearity, in Figure 5, we display the density of nonlinear stationary states as a function of nonlinearity χ and the percentage of LR connections, $B_{\mathrm{per}}$. The square-root singularity distribution is dominant until the percentage of additional connections reaches the critical value, $B_{\mathrm{per}}^{c}\approx 3\%$, while the nonlinearity breaks the symmetry of the spectrum. For $B_{\mathrm{per}}>B_{\mathrm{per}}^{c}$, the frequency spectrum has a more semicircular profile. Close to the MF limit, the new stationary states are concentrated more at the center for all values of nonlinearity.

For a more quantitative description of the localization of wavefunctions, we consider a single measure as the “localization index”. A commonly used method to assess the degree of localization is the so-called “participation ratio”, or a similarly defined quantity referred to as the “inverse participation ratio” [7,36]. The localization index, α, we will use is defined as

(10) $$\begin{array}{c} \alpha =\sum_{j=1}^{n}{\left|A_{j}\right|}^{4}\;\times {\left(\sum_{j=1}^{n}{\left|A_{j}\right|}^{2}\right)}^{-2} \end{array}$$

where $A_{j}$ are the amplitudes of the stationary states of the system. The localization index α reaches its minimum value of 1/n when the wavefunction is evenly distributed between all sites, indicating a completely delocalized state. In contrast, the localization index reaches its maximum value of unity when the wavefunction is completely concentrated at a single site, which signifies perfect localization. In order to have a picture of the overall system localization, we use a heuristic criterion and classify the states as “localized” if α≥0.5 and “delocalized” if α<0.5. This distinction is not abrupt and the term is certainly not used in a very rigorous manner. It helps, however, in obtaining a general qualitative picture that complements the quantitative results.

**Figure 6:**
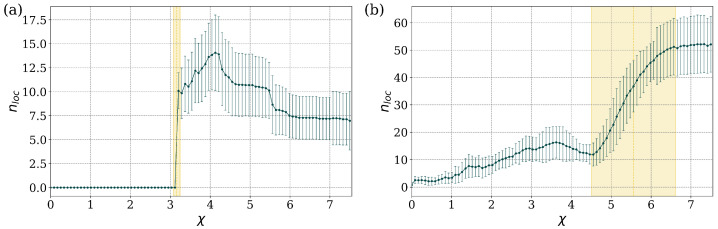
Average number of localized states, $n_{loc}$, as a function of nonlinearity χ: (**a**) $B_{\mathrm{per}}=0.06\%$ and (**b**) $B_{\mathrm{per}}=97.01\%$, for the system with γ=1.0. A transition to enhanced localization for χ values larger that those in a certain critical regime is apparent; the critical regime is (**a**) $\chi^{c}\approx 3.16\pm 0.09$, and (**b**) $\chi^{c}\approx 5.56\pm 1.05$.

**Figure A1:**
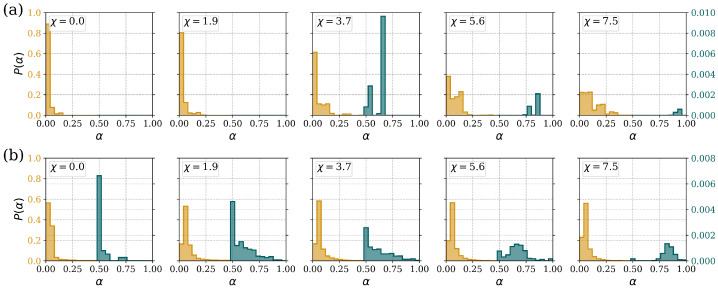
Probability distribution P(α) of states as a function of the localization index α for the system with equal strength bonds, γ=1.0, and fraction of LR connections: (**a**) $B_{\mathrm{per}}=0.06\%$, and (**b**) $B_{\mathrm{per}}=97.01\%$. These results correspond to the DOS in Figure 4. The orange color indicates the delocalized states (α<0.5), while the green color denotes the localized states (α≥0.5).

We examine in some detail two illustrative cases, near the NN limit and near the MF limit. In the first case, near the NN limit ($B_{\mathrm{per}}=0.06\%$), and for the linear regime, χ=0, all the states are delocalized. As χ increases beyond a critical value, $\chi^{c}\approx 3.16$, localized states begin to appear and the average number of these states, denoted as $n_{loc}$, initially increases with χ and eventually settles to an approximately constant value, lower than the maximum value. This behavior is captured in Figure 6a. The degree of localization (value of α) also grows with increasing values of χ beyond the critical value. A more detailed picture for several values of χ is provided in Appendix A, Figure A1a.

In the second case, near the MF limit ($B_{\mathrm{per}}=97.01\%$) Figure 6b, there are localized states for all values of χ, and their average number increases approximately linearly with χ, until a critical range is reached for $\chi^{c}\approx 5.56\pm 1.05$. In this range, the average number of localized states increases rapidly until it reaches an approximately constant value. A more detailed picture for several values of χ can be found in Appendix A, Figure A1b. The parameter χ determines the amount of nonlinearity that is represented in the DNLS model. If the nonlinearity is small, the system is quasi-linear and all stationary states are delocalized. As the nonlinearity increases, there is a sudden appearance of localized states, which is observed both in the stationary solutions and in the underlying dynamical evolution of the system [12,17].

**Figure 7:**
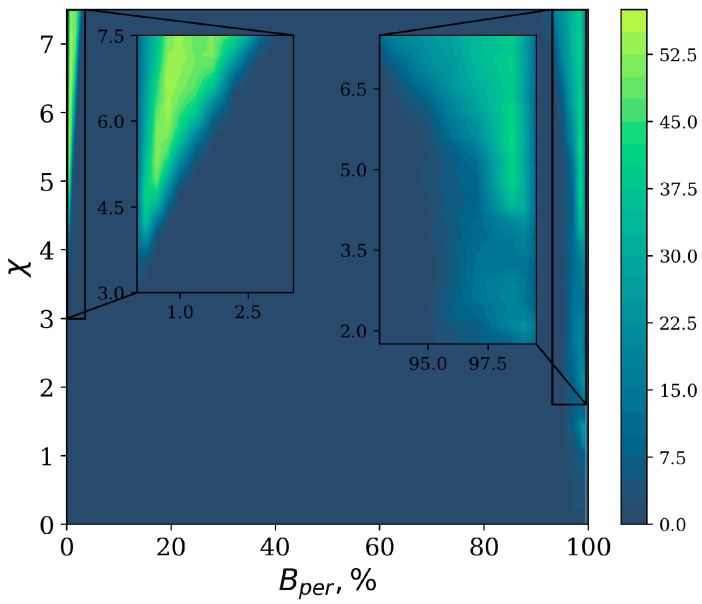
Phase diagram of localized stationary states as a function of nonlinear parameter χ and percentage of LR connections $B_{\mathrm{per}}$, for γ=1.0. The color-coding represents the average number of localized states $n_{loc}$. The insets show an expanded version of the state localization–delocalization features.

A phase diagram of the regions in the $\left(\chi ,B_{\mathrm{per}}\right)$ plane of localized and delocalized states is shown in Figure 7. The color map indicates the average number of localized states, $n_{loc}$, obtained from 10 unique constructions of the matrix *M* defined in Equation (6). Localization is observed exclusively in the vicinity of the NN limit and the MF limit; once the percentage of additional long-range connections exceeds $B_{\mathrm{per}}∼3\%$, the localized states disappear, and they only appear again for large percentages, $B_{\mathrm{per}}\geq 93\%$. It is interesting that in the vast intermediate regime, practically no localized states exist for the choice of parameter values considered here. In particular, the transition from a square-root singularity to a semicircular distribution in the spectrum coincides with the $B_{\mathrm{per}}^{c}∼3\%$ of the LR connections.

**Figure 8:**
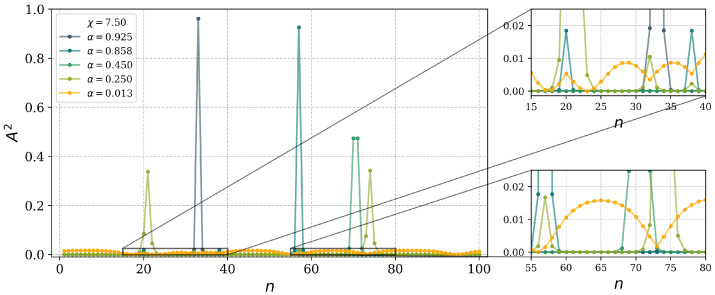
Distribution of the states over a lattice of n=100 sites, for non-linearity χ=7.5 and LR bonds $B_{\mathrm{per}}=0.06\%$. States of various degrees of localization (values of α, corresponding to different color lines) are shown.

It is also of interest to consider the degree of localization and the distribution of localized states throughout the system. An illustrative example is shown in Figure 8. In this case, in a system of n=100 sites and for $B_{\mathrm{per}}=0.06\%$, the localized stationary states exhibit a relatively uniform distribution throughout the lattice, with minor fluctuations (orange line for α=0.013). The delocalized states with localization indices α=0.25 (green line) and α=0.45 (cyan line) demonstrate shallow localization peaks, while their wavefunctions are spread evenly across the system. In contrast, fully localized states with α=0.858 (blue line) and α=0.925 (grey line) display a δ-function-like distribution, with the localization concentrated at a single site.

### 3.3. Random Connections with Unequal Strength Bonds

**Figure 9:**
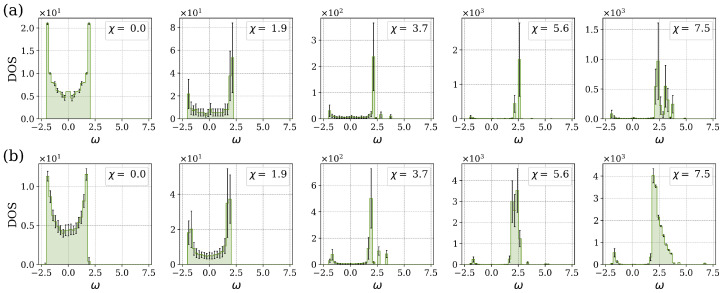
The DOS as a function of frequency ω and different values of the nonlinear parameter χ, for (**a**) $B_{\mathrm{per}}=0.06\%$, and (**b**) $B_{\mathrm{per}}=97.01\%$, in a system with n=100 sites and γ=0.1. The error bars represent the standard deviation.

We also considered a model in which the strengths of the NN and LR connections were not equal. Specifically, we studied the case γ=0.1 (LR bond strength is one-tenth of the NN bond strength). This can be interpreted as a distance-dependent connectivity, with all neighbors beyond the nearest one having a much reduced strength. The resulting DOS for various values of the nonlinearity parameter χ is presented in Figure 9.

As in the case of γ=1.0 (see Figure 4), the DOS for the system with reduced LR connection strengths for $B_{\mathrm{per}}=0.06\%$ displays the distinctive square-root singularity, characterized by sharp peaks in the nonlinear stationary states near ω=±2.5, with an almost smooth transition between these peaks, as illustrated in Figure 9a. The nonlinear parameter χ modifies the symmetry of the DOS spectrum, leading to an increased concentration of states at the edge of the right band of the spectrum. Furthermore, the right peak is more pronounced as the nonlinearity parameter χ is increased.

For the system with $B_{\mathrm{per}}=97.01\%$, the DOS spectrum exhibits the same characteristics as the NN case, as seen in Figure 9b. The strong NN bond strength, which is ten times greater than that of the LR bonds, leads to the excitation propagating preferentially through the NN connections. This results in the NN spectrum dominating the overall DOS. The peaks in the DOS spectrum are located at the eigenvalues ω=±2, and the concentration of states increases on the right side of the spectrum as a function of the nonlinearity. The transition in the shape of the DOS from a square-root singularity to the semicircular profile is expected to occur at the critical percentage of LR connection of $B_{\mathrm{per}}^{c}=50\%$ when γ=0.1, as in the linear case. Furthermore, the distribution of the DOS exhibits symmetry when moving from the $B_{\mathrm{per}}^{c}$ towards the NN and MF limits, as previously demonstrated in the linear case [5].

**Figure 10:**
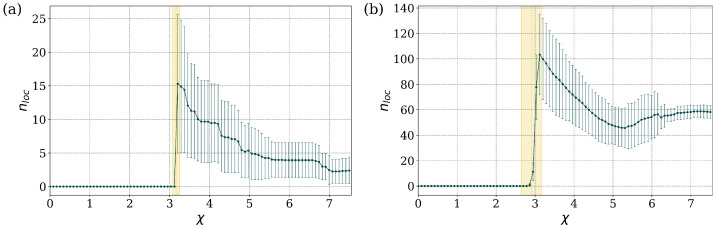
Average number of localized states, $n_{loc}$, as a function of nonlinearity χ: (**a**) $B_{\mathrm{per}}=0.06\%$ and (**b**) $B_{\mathrm{per}}=97.01\%$, for the system with γ=0.1. A transition to enhanced localization for χ values larger that those in a certain critical regime is apparent; the critical regime is (**a**) $\chi_{c}\approx 3.16\pm 0.04$, and (**b**) $\chi_{c}\approx 2.91\pm 0.25$.

**Figure A2:**
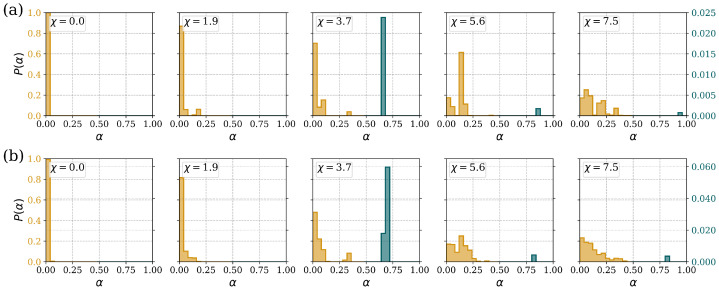
Probability distribution P(α) of states as a function of the localization index α for the system with equal strength bonds, γ=0.1, and fraction of LR connections: (**a**) $B_{\mathrm{per}}=0.06\%$, and (**b**) $B_{\mathrm{per}}=97.01\%$. These results correspond to the DOS in Figure 9. The orange color indicates the delocalized states (α<0.5), while the green color denotes the localized states (α≥0.5).

We performed the analysis based on the localization index, using similar steps as for the case γ=1.0 discussed earlier. Detailed examples of the behavior of the localization index are presented in Appendix A, see Figure A2. The overall behavior is captured in Figure 10, which presents the average number of localized states, $n_{loc}$, as a function of the parameter χ. For both the NN limit ($B_{\mathrm{per}}=0.06\%$) and the MF limit ($B_{\mathrm{per}}=97.01\%$), the states are completely delocalized and uniformly distributed across the lattice in the absence of nonlinearity (χ=0). The localized states only emerge when the nonlinearity parameter χ exceeds the critical value of $\chi^{c}\approx 3.0$. For $\chi >\chi^{c}$, the average number of localized states decreases as the nonlinearity increases until it reaches an approximately constant value. The localization index for these systems mirrors closely the behavior observed in the DOS distribution of systems, in which γ=1.0, in the NN limit (see Appendix A).

## 4. Conclusions

Motivated by the intriguing behavior of moiré layered systems, where flat bands and strong particle interactions conspire to give rise to complex correlated states, we studied the discrete nonlinear Schrödinger (DNLS) system in a small-world network. This system contains, in a simple manner, all the important ingredients of interest, namely disorder (in the randomness of the small-world network), long-range interactions, and nonlinearity-induced correlations. The DNLS system is a fundamental nonlinear equation which has many applications in condensed matter physics and optics. It is formulated as a discrete set of equations that is not solvable analytically except for some simple cases; the general case requires the use of numerical techniques. The DNLS equation has a stationary form that is similar to the eigenvalue form of a linear equation, with the spectrum of its stationary values being similar to the frequency of a corresponding linear spectrum. Analyzing the nonlinear modes of DNLS in a small-world network provides information on the interplay of nonlinearity and disorder with long-range interactions. The model we focused on in this work covers the range from the nearest neighbor (NN) DNLS equation system to the fully-connected, mean field (MF) limit. In this progression, the modes lose the typical character of the NN limit and acquire a more MF-like character. In the latter case, while all lattice sites are coupled to each other, the wavefunctions are not necessarily extended but may include localized features. This aspect is also present in the *linear* small-world tight-binding model [5]. The presence of nonlinearity provides and additional localization agent, even at the sparse long-range bond limit.

Near the NN limit, a sequence of new solutions papering in the stationary spectrum introduces new states as a function of nonlinearity. The statistical analysis of these states shows that their distribution shifts towards the linear band edge with the nonlinearity. As the number of long-range bonds increases, the spectrum of states changes character and becomes similar to a semicircular shape, while moving towards the band center. We note that the dependence of the spectrum on the nonlinearity and long-range bond number, while complex, fundamentally exhibits a simple pattern: it evolves towards the band edge with increased nonlinearity, and towards the band center with an increasing number of long-range bonds. This behavior corresponds to the case where NN and LR bonds have the same value, while when LR bonds are weaker than NN bonds, the spectrum is naturally dominated by features related to the NN limit. The transition between the two limits occurs at a critical value of additional long-range bonds. During this transition, the stationary states enhance their localization character. We found that, close to the MF limit, the localization character of the stationary states is also more dominant in the presence of nonlinearity. In general, nonlinearity enhances localization in the MF limit, while the energy eigenvalues of the states are distributed around zero-energies. Nonlinearity flattens the band and introduces additional correlations in the available states.

In the original theoretical work on tBLG, it was observed that, while twist angles larger than a few degrees lead to largely electronically isolated layers, when the twist angles become smaller, the interlayer coupling strengthens, leading to decreased quasiparticle velocity at the Dirac point and thus to a flat band [3]. By analogy, small-world geometry leads to a flat band limit when the number of bonds is close to the fully coupled mean field limit [5]. Setting aside the fact that the model studied here is one-dimensional in space, one may associate the moiré length scale in twisted bilayer graphene to the parameter $B_{\mathrm{per}}$ of the present work, since they appear to play analogous roles in defining the electronic structure of their respective systems, particularly in mediating the transition between localized and delocalized states. In twisted bilayer graphene, the moiré length controls band flattening and localization, while in the SW-DNLS model, $B_{\mathrm{per}}$ determines the spectral shape and degree of state localization. While the present model gives a descriptive behavior of the role of LR bonds in the presence of correlations, it is necessary to derive a more quantitative picture of the connection of the moiré lattices with reduced long-range nonlinear lattice models. Furthermore, the use of machine learning tools for this problem may make it possible to differentiate the nature of the states, but also look deeper into the connection of the model with the physical moiré structures [37].

## Data Availability

The original contributions presented in the study are included in the article, further inquiries can be directed to the corresponding author.

## Abbreviations

The following abbreviations are used in this manuscript:

tBLG	twisted bilayer graphene
DNLS	Discrete Nonlinear Schrödinger
SWN	Small-World Network
SW-DNLS	Small-World Discrete Nonlinear Schrödinger system
DOS	Density Of States
LR	long-range interactions
NN	Nearest Neighbor
MF	Mean Field

## Appendix A. Localization Index

We show examples of the behavior of the localization index defined in Equation (10) for the cases of γ=1.0, Figure A1, and γ=0.1, Figure A2. In each case, we use two values for the percentage of LR bonds: one close to the NN limit ($B_{\mathrm{per}}=0.06\%$) and the other close to the fully-connected MF limit ($B_{\mathrm{per}}=97.01\%$).

The frequencies of stationary states were found by numerically solving the system of *n* nonlinear equations, Equation (6). We chose a system with n=100 sites, which had a maximum of $B_{max}=4850$ LR connections. The initial eigenvalues with zero nonlinearity χ=0 were calculated analytically [6]. The nonlinearity parameter was allowed to take values in the range of 0.0 to 7.5 in steps of 0.085. By incrementally increasing the nonlinear parameter, the subsequent nonlinear stationary states could be obtained through continuation methods [13,18,19,20,21,22]. Once the nonlinear stationary states had been determined for a specific value of the nonlinear parameter, these solutions served as initial approximations for the next parameter value. The bifurcation points were identified by analyzing the Jacobian function. The isolated branch of the frequency diagram can be found by using the continuation method with a perturbation, deflation technique, or two-parameter continuation [38]. Using the arc-length continuation method with a small perturbation, additional initial guesses are introduced by applying small perturbations to previous solutions to search for new stationary solutions within a region near a known point. This method follows the continuation path to locate new/isolated branches [39]. A perturbation can be added to each true initial solution. This creates a set of additional possible solutions based on the true initial condition, but with a small variation in the perturbation. Each of these solutions will collapse to a known state, which will not be counted again. Some of the new solutions will fall into the unknown branch. The deflated continuation method extends Newton’s method by using a deflation operator to eliminate previously found solutions, allowing the discovery of isolated branches in bifurcation diagrams [40,41,42]. All calculations were performed using Python.

The stability of the stationary solutions is determined through a standard linear stability analysis, following the approach outlined in [4]. This method involves the introduction of small time-dependent perturbations around a stationary state ψ(t)=exp(iωt)(A+u(t)). Substituting the updated stationary states into the DNLS Equation (3) and linearizing with respect to u(t) leads to a system of linear differential equations from which the corresponding eigenvalue problem can be derived. The perturbation is generally expressed as $A=A_{1}+iA_{2}$. If all eigenvalues of the coefficient matrix have real parts <0, the solution is considered to be stable. In contrast, the sufficient condition for the unstable state is that all the eigenvalues have real positive parts. This method contributes to the identification of stable and unstable branches within the solution space.

Using the computed eigenvectors *A*, the localization index was calculated based on Equation (10) and states were classified as localized if α≥0.5 and as delocalized otherwise. For each value of χ, we constructed a sample of 35 solutions from which the average number of localized states $n_{loc}$ and the standard deviation were computed. The values of $n_{loc}$ as a function of χ (with error bars equal to the standard deviation) are shown in Figure 6 and in Figure 10 for different values of the LR bond strength, γ=1.0 and γ=0.1, respectively.
